# Comparable Overall Copulation Rates Yet Rank‐Biased Access to Likely Fertile Females in Male Bonobos at Wamba

**DOI:** 10.1002/ajpa.70318

**Published:** 2026-07-16

**Authors:** Kazuya Toda, Furuichi Takeshi

**Affiliations:** ^1^ Research Center for Integrative Evolutionary Science The Graduate University for Advanced Studies, SOKENDAI Hayama Japan; ^2^ Sugiyama Jogakuen University Aichi Japan

**Keywords:** intermale mating competition, non‐conceptive copulation, operational sex ratio, *Pan paniscus*, prolonged sexual receptivity

## Abstract

**Objectives:**

Female bonobos ( *Pan paniscus* ) resume sexual swellings in the early postpartum stage and exhibit prolonged sexual receptivity. Despite the simultaneous availability of receptive females, genetic evidence reveals a pronounced male reproductive skew in this species. We investigated how male bonobos allocate mating effort to females in the likely fertile window (LFW) versus non‐conceptive phases based on their dominance rank and dynamic party‐level competitive situations.

**Materials and Methods:**

We conducted all‐day focal animal sampling on adult and adolescent bonobos in a free‐ranging group at Wamba. Female fertile windows were estimated using the end of the maximal swelling phase. Generalized linear mixed models were used to analyze rank‐related mating patterns.

**Results:**

Overall copulation rates did not differ across male dominance ranks. However, higher‐ranking males secured a disproportionate share of copulations with LFW females. At the party level, higher‐ranking males preferentially copulated with LFW females when present, whereas lower‐ranking males were more likely to copulate with non‐LFW females as the number of LFW females increased.

**Discussion:**

Our findings add evidence that the pronounced male reproductive skew in bonobos is driven by rank‐biased access to fertile females rather than total mating frequency. Subordinate males likely redirect mating effort toward receptive but non‐conceptive females rather than directly contesting with dominants over fertile females or forming consortships. This flexible shift in mating targets represents an alternative tactic that allows subordinates to marginally increase their participation in sperm competition despite high monopolization by dominants or to develop affiliative relationships with numerous females for future mating advantages.

## 1 Introduction

Sexual receptivity refers to the physiological, morphological, and behavioral readiness of a mammalian female to accept a male and engage in copulation (Dixson 1998). In most non‐primate mammals, sexual receptivity is largely limited to the periovulatory phase of the estrous cycle and tightly regulated by fluctuations in ovarian hormones such as estrogen and progesterone (Conaway 1971; Weir and Rowlands 1973). Likewise, strepsirrhine primates exhibit sexual receptivity only within a relatively narrow fertile window (Small 1988). In contrast, anthropoid primates can remain receptive outside the fertile window (Wallen and Zehr 2004). However, this extended period of sexual receptivity can impose fitness costs, including opportunity and energetic costs associated with sexual activity (Kunz et al. 2021), greater exposure to male harassment and coercion potentially resulting in injury (Muller et al. 2011), and an increased risk of sexually transmitted pathogens (Phillips‐Conroy et al. 1994). Nevertheless, such costs may be outweighed by adaptive benefits, such as promoting paternity confusion to reduce the risk of male infanticide or eliciting paternal investment to enhance offspring survivorship (Fernandez‐Duque et al. 2020; Rooker and Gavrilets 2020).

Across anthropoids, females vary widely in the extent and manner in which they advertise fertility to males. In some polygynandrous taxa, females express graded visual cues of ovulation, such as sexual skin tumescence or skin coloration, to bias male mating effort toward fertile periods. By maintaining these signals over a prolonged period of sexual receptivity, females can increase the probability of conception while also promoting paternity confusion (e.g., Engelhardt et al. 2004; Setchell and Wickings 2004; Gesquiere et al. 2007; Dubuc et al. 2009; Higham et al. 2009). In monogamous taxa, by contrast, females typically exhibit subtle or absent visual ovulatory cues (i.e., concealed ovulation) while remaining sexually receptive over extended phases of their ovulatory cycles (e.g., Kendrick and Dixson 1983; Barelli et al. 2008; Fernandez‐Duque et al. 2011). However, the relationship between concealed ovulation and mating systems is not strictly dichotomous. For example, a comparative phylogenetic analysis suggests an evolutionary scenario in which concealed ovulation among anthropoids likely originated under polygamous or polygynandrous mating systems as a counterstrategy against male infanticide (Sillén‐Tullberg and Møller 1993).

Chimpanzees ( *Pan troglodytes* ) and bonobos ( *Pan paniscus* ) are prominent examples of the polygynandrous anthropoids with visual ovulatory cues. Characterized as non‐seasonal breeders with interbirth intervals of approximately 4–6 years, both species live in multi‐male/multi‐female groups with promiscuous mating systems, in which female sexual skin tumescence closely tracks fluctuating estrogen and progesterone levels (Graham et al. 1972; Heistermann et al. 1996; Emery Thompson and Sabbi 2024). Because other hominids—including orangutans, gorillas, and humans—exhibit subtle or absent ovulatory cues, this conspicuous advertisement of sexual receptivity likely evolved in the *Pan* ancestor after divergence from the hominin lineage (Sillén‐Tullberg and Møller 1993). Furthermore, both female chimpanzees and bonobos exhibit prolonged sexual receptivity, as evidenced by maximal sexual skin tumescence that persists beyond the fertile window and occurs post‐conception and during the postpartum infertile phase (Deschner et al. 2003; Douglas et al. 2016).

Nonetheless, the overall period of prolonged sexual receptivity is substantially more extensive in bonobos than in chimpanzees (Furuichi 2011). This interspecific difference is driven primarily by two reproductive parameters: a maximal swelling phase that averages approximately 2 days longer in bonobos (13.5 ± 1.8 days) than in chimpanzees (11.3 ± 1.1 days) (reviewed by Ryu et al. 2025), and a markedly compressed interval between parturition and the resumption of sexual skin tumescence. Whereas female bonobos resume sexual swellings at a mean of 0.7 ± 0.4 years postpartum (Hashimoto et al. 2022), this resumption is delayed significantly in chimpanzees, occurring at 1.8–2.4 years in the western subspecies (*P. t. verus*: Deschner and Boesch 2007) and 3.9–4.6 years in eastern subspecies populations (*P. t. schweinfurthii*: Wallis 1997; Nishida et al. 2003; Emery Thompson and Ellison 2017). This accelerated postpartum resumption exerts a more profound influence on the total receptive timeframe in bonobos than does the incremental lengthening of each ovulatory cycle (Furuichi 2011).

The priority‐of‐access (PoA) model (S. A. Altmann 1962) posits that males queue for mating opportunities based on their competitive ability and the simultaneous availability of receptive females. According to this model, the most dominant male can exclusively monopolize mating success when only a single female is receptive, but this monopolization breaks down when multiple females become receptive concurrently (Bulger 1993; Weingrill et al. 2000; Alberts et al. 2003; Boesch et al. 2006; Ostner et al. 2008). Furthermore, an increase in the number of male rivals can cause dominant males to lose complete control over mating access due to the escalating costs of mate guarding (Alberts et al. 2003; Kutsukake and Nunn 2006; Weir et al. 2011). Building on this theoretical framework, Furuichi (2011) hypothesized that the exceptionally early resumption of postpartum swelling in female bonobos increases the simultaneous availability of receptive females, thereby preventing a few dominant males from monopolizing mating success. Indeed, the average ratio of receptive females to mature males is lower in bonobo groups at Wamba than in chimpanzee groups at Mahale and Gombe (2.8 vs. 4.2 and 12.3; Furuichi and Hashimoto 2002). Accordingly, these dynamics led researchers to expect a substantially lower male reproductive skew in bonobos than in chimpanzees.

However, recent genetic paternity data from multiple bonobo populations (LuiKotale, Wamba, and Kokolopori) consistently reveal a pronounced male reproductive skew (Surbeck, Langergraber, et al. 2017; Ishizuka et al. 2018; Mouginot et al. 2023). The most successful male bonobos sire a significantly larger share of offspring than observed in male chimpanzees (69% ± 11% vs. 44% ± 25%; Mouginot et al. 2023). Male reproductive success strongly correlates with dominance rank: for example, alpha males achieved the highest reproductive success (Ishizuka et al. 2018) and sired more offspring than expected at Kokolopori (Mouginot et al. 2023). These genetic findings indicate that dominant males secure a disproportionate share of fertilizations despite the ambiguity of ovulatory signals caused by prolonged sexual swellings, including those during postpartum infertile periods. Furthermore, behavioral and hormonal data demonstrate that males preferentially direct their mating effort toward females during the fertile window (Ryu et al. 2025). Coupled with the power of dominant males to control subordinates' mating access (Kano 1996; Furuichi 1997; Hohmann and Fruth 2003; Surbeck et al. 2012; Yokoyama and Furuichi 2022), males' cognitive capacity to extract usable information from probabilistic ovulatory signals helps explain the proximate mechanisms underlying the pronounced reproductive inequality in this species (Ryu et al. 2025).

Female sociality may fundamentally shape this high male reproductive skew in bonobos through three interconnected pathways: spatial cohesion, active mate choice, and maternal support. Although bonobos exhibit dynamic fission‐fusion grouping patterns (Kuroda 1979; White 1988), females maintain high party‐level associations (Furuichi 1989; White 1992; Hashimoto and Furuichi 2015; Surbeck, Girard‐Buttoz, et al. 2017). High female spatial cohesion may enable dominant males to efficiently monitor female reproductive states and activity (e.g., red deer, *Cervus elaphus* : Clutton‐Brock et al. 1982; elephant seals, *Mirounga* spp.: Hoelzel et al. 1999). Furthermore, female–female agonistic coalitions elevate female dominance status above that of males (Parish 1996; Tokuyama and Furuichi 2016; Surbeck et al. 2025), granting females the leverage to refuse unwanted courtship and sexual coercion (Furuichi and Hashimoto 2004; Paoli 2009). If females rely on male dominance rank as a proxy for genetic quality (e.g., Kirkpatrick 1996; Møller and Alatalo 1999), female mate choice might further concentrate reproductive success toward dominant males. Moreover, maternal presence drives male reproductive success; mothers facilitate their sons' access to receptive females and provide agonistic support during conflicts with male rivals (Furuichi 1997; Surbeck et al. 2011; Surbeck, Langergraber, et al. 2017). High maternal dominance rank further amplifies this reproductive advantage, consolidating the sons' monopolization of fertile females (Yokoyama and Furuichi 2022; Shibata and Furuichi 2023).

A central unresolved question is how the increased concurrent availability of maximally tumescent females, driven by prolonged sexual receptivity, shapes the rank‐related mating patterns of male bonobos. When competitive exclusion blocks access to females with high conception probabilities, subordinate males face three tactical options: (i) engage in direct contests with dominants, (ii) employ opportunistic sneaking tactics, or (iii) redirect their mating efforts toward females with lower conception probabilities. The first option carries a risk of injury, and its success occasionally requires agonistic maternal support to overcome dominant males, who monopolize mating access with the backing of their dominant mothers. However, because female dominance status in bonobos typically increases with age (Furuichi 1997; Vervaecke et al. 2000; Tokuyama and Furuichi 2016; Toda and Furuichi 2020), younger mothers seldom overpower older mothers (Shibata and Furuichi 2023), limiting the conditions under which this tactic succeeds. The execution of the second option is constrained by high female spatial cohesion, which enables dominants to effectively control access to mates. Indeed, bonobos seldom form consortships—temporary, exclusive mating associations away from other group members (Shibata and Furuichi 2023)—unlike chimpanzees (Tutin 1979; Matsumoto‐Oda 1999; Constable et al. 2001; Wroblewski et al. 2009; Newton‐Fisher et al. 2010; Bray et al. 2016). The third option remains readily available to subordinate males because bonobo groups frequently contain multiple receptive females. Although ejaculatory investment in females with lower conception probabilities rarely translates into paternity, it can marginally increase opportunities for sperm competition. Such a “best‐of‐a‐bad‐job” tactic of subordinate males is widely observed across anthropoids (e.g., chimpanzees: Constable et al. 2001; Japanese macaques, *Macaca fuscata* : Soltis et al. 1997; long‐tailed macaques, *Macaca fascicularis* : Engelhardt et al. 2006; yellow baboons, *Papio cynocephalus* : Alberts et al. 2003).

To examine how male bonobos allocate mating effort toward females with lower conception probabilities in response to their dominance status and dynamic party‐level competitive situations, we investigated mating patterns in a free‐ranging bonobo group at Wamba. Specifically, using all‐day focal animal sampling on adult and adolescent individuals and an empirical method to estimate a likely fertile window (LFW) for females based on the end of the maximal swelling phase (MSP), we tested the following three predictions:

1. Males will exhibit comparable overall copulation rates across the dominance hierarchy. If receptive females with lower conception probabilities attract males and engage in copulations, mating opportunities should become available to a broader range of males when multiple receptive females are present simultaneously. We tested this prediction by examining the effect of male rank on overall copulation rates using male focal data.
2. Dominant males will monopolize access to fertile females. Given the pronounced male reproductive skew, dominant males should account for a disproportionate share of copulations with females during the fertile window. We tested this prediction by comparing the proportion of copulations involving higher versus lower‐ranking males between LFW and non‐LFW females using female focal data.
3. Subordinate males will redirect mating effort toward females with lower conception probability under competitive constraints. If males obtain mating opportunities by targeting receptive females that remain accessible given their respective competitive ability, subordinates would be more likely than dominants to copulate with non‐LFW females, specifically when LFW females are concurrently present in the party. Using male focal data, we tested this prediction by analyzing how within‐party male rank affects the probability of copulation with non‐LFW females, controlling for the number of LFW females.

## 2 Materials and Methods

### 2.1 Study Group and Subjects

This study was conducted on a single habituated group of free‐ranging bonobos (the E1 group) at Wamba in the Luo Scientific Reserve, Democratic Republic of the Congo, a site that has been continuously studied since 1974 (Kano 1992; Furuichi et al. 2012). Observational data were collected over 153 days, from December 1, 2022, to May 2, 2023. During this period, the group comprised 45–48 individuals: 10 adult and two adolescent males, alongside 17 adult and three adolescent females. Group size has increased gradually since 2009, primarily due to the immigration of nulliparous females (Toda et al. 2023), resulting in the highest recorded number of adult and adolescent females to date (Figure S1). All adult and adolescent females were immigrants. Among the males, all but two (DI and GC) were natal; eight of them co‐resided with their mothers.

Age classes were defined by sex‐specific developmental and reproductive milestones:

1. Adult males (≥ 12 years): Corresponding to the attainment of maximum body mass in captive males (Berghaenel et al. 2023).
2. Adolescent males (8 to < 12 years): Encompassing the marked rise in urinary testosterone around 7 years of age (Behringer et al. 2014; Berghaenel et al. 2023) and the earliest recorded reproduction in captivity (Thompson‐Handler 1990).
3. Adult females (≥ 12 years or parous): Aligning with the mean age at first reproduction at Wamba (11.7 ± 1.6 years; Toda et al. 2023).
4. Adolescent females (nulliparous, 8 to < 12 years): Reflecting the mean age of natal dispersal (7.1 ± 0.5 years) and increased copulatory activity post‐immigration (Toda et al. 2022).

We conducted all‐day focal animal sampling on all adult and adolescent individuals in the E1 group (Table 1: males; Table 2: females), with three exceptions. One adult male (TN) was excluded due to prolonged absence until March 6, 2023; one adult female (Aq) was also excluded because insufficient habituation precluded undisturbed focal follows; and another adult female (No) yielded only a single all‐day focal follow prior to her final sighting on December 31, 2022.

**TABLE 1:** Basic information about male subjects in the E1 group (December 2022–May 2023).

ID	Age^a^ (years)	Resident mother^b^	Within‐sex dominance rank [David score]	Focal observation hours and days	Counts of copulation events during focal follows	
					With adults	With adolescents
TN^c^	52*	—	Not used [−2.9]	Not collected	—	—
TW^d^	49*	—	Not used [−12.9]	39.2 h (4 days)	0	0
DI	48*	—	5th [4.6]	39.7 h (4 days)	4	3
NB	35	Ki	4th [7.2]	44.7 h (5 days)	5	4
GC	33*	—	8th [−11.2]	37.5 h (4 days)	3	4
KT	19	Ki	1st [53.4]	36.4 h (4 days)	3	0
JO	17	Jk	3rd [7.5]	36.7 h (4 days)	4	7
KY	14	Ki	2nd [10.0]	30.6 h (4 days)	5	1
HC	14	Hs	6th [−2.7]	32.0 h (4 days)	5	1
SE	12	Sl	7th [−6.1]	32.6 h (4 days)	7	2
NI	10	Nv	10th [−28.9]	25.1 h (4 days)	5	2
YD	9	Yk	9th [−27.0]	34.4 h (4 days)	0	2

*Note:* Data in parentheses were not used for the analysis.

^a^ Asterisks in the column mean estimated ages.

^b^ See Table 2.

^c^ TN was not included as a focal subject because he had been lost for 5 months in the E1 group and was absent at the beginning of this study.

^d^ TW had seemed to have erectile dysfunction before the study period, and thus, data on him were not used for the analysis.

**TABLE 2:** Basic information about female subjects in the E1 group (December 2022–May 2023).

ID	Age (years)^a^	Within‐sex dominance rank [David score]	Length after last parturition (months)^b^	Number of maximal swelling phases	Number of likely fertile periods	Focal observation hours (and days)
No^c^	52*	12th [−2.3]	92	1	0	9.0 h (1 day)
Ki	49*	1st [41.5]	43	3	3	38.0 h (4 days)
Hs	42*	2nd [10.2]	30	4	2	40.7 h (4 days)
Yk	40*	4th [5.3]	16	1	0	35.7 h (4 days)
Jk	35*	9th [−0.29]	28	4	3	38.1 h (4 days)
Sl	31*	6th [4.7]	22	3	2	35.9 h (4 days)
Nv	29*	8th [2.8]	5	0	0	38.3 h (4 days)
Ot	26*	10th [−0.96]	10	3	0	34.5 h (4 days)
Fk	25*	5th [4.8]	33	3	3	36.2 h (4 days)
Pf	19*	3rd [5.3]	25	3	3	35.9 h (4 days)
Ik^d^	16*	15th [−6.0]	26	0	0	34.5 h (4 days)
Sc^d^	15*	11th [−1.2]	44	0	0	29.5 h (4 days)
Db	14*	7th [4.1]	35	3	0	27.1 h (3 days)
Jj	12*	17th [−10.1]	6	2	0	38.3 h (4 days)
Aq	12*	13th [−2.4]	4	4	0	Not collected
Ec^d^	11*	14th [−4.0]	—	0	0	38.6 h (4 days)
Mz^e^	10	18th [−10.8]	6	3	1	36.5 h (4 days)
Tp	10*	20th [−11.7]	—	5	0	35.5 h (4 days)
Bc	10*	19th [−11.3]	—	2	0	33.0 h (4 days)
Ih	9	16th [−8.9]	—	4	0	34.2 h (4 days)

^a^ Asterisks in the column mean estimated ages.

^b^ At the beginning of this study period (December 6th, 2022).

^c^ No disappeared from E1 after December 31, 2022, and is presumed dead due to old age.

^d^ Ik, Sc, and Ec were pregnant and gave birth to their infants during the study period.

^e^ Mz gave birth to her first infant at 9.6 years of age but lost it at 4 months after parturition.

### 2.2 Protocol of All‐Day Focal Animal Sampling

A team of researchers and trained local assistants followed a single party (i.e., sub‐group) of the E1 group daily. Observations usually started when the bonobos descended from their night beds at approximately 0600 h and ended when they constructed new night beds around 1700 h. The first author (KT) and research assistant Lokemba Batsindelia (LB) conducted continuous focal animal sampling (J. Altmann 1974) on adults and adolescents to record behavioral data and party composition through the day.

On each observational day, we selected a focal subject using a predetermined random sequence. If the assigned individual could not be located at the party within 30 min, we selected the next available subject. A focal follow continued until the subject constructed a night bed, darkness after sunset precluded identification, or the subject remained out of sight for ≥ 2 consecutive hours. If the subject was out of sight for ≥ 2 consecutive hours before reaching 4 h of observation, we terminated the focal follow and initiated a second focal follow on a different individual, provided it could begin by 1200 h. Only focal data with at least 4 h of observation were retained as “all‐day” focal data. To balance observation effort, we prioritized undersampled subjects midway through the study and ensured a minimum 20‐day interval between resampling the same individual.

During each focal follow, we recorded states and events—including resting, feeding, moving, and social interactions—alongside their durations and the participants involved. Following the one‐hour party method (Hashimoto et al. 2001), we defined party composition as the set of adult and adolescent individuals present within fixed hourly blocks (e.g., 0600–0700 h, 0700–0800 h, and so forth up to 1600–1700 h). We excluded party data outside the 0600–1700 h range to ensure reliable visibility. Upon temporarily losing a focal subject, we paused data collection and resumed only upon successful relocation.

In total, we collected 1033.8 h of all‐day focal data over 116 days (KT: 544.8 h; LB: 489.0 h). This dataset included 384.1 h across 45 days for 11 males (Table 1) and 649.7 h across 71 days for 19 females (Table 2). Daily focal observation time averaged 8.9 ± 1.4 h (range: 4.2–11.0 h), with median start and end times of 0631 h (range: 0551–1142 h) and 1700 h (range: 1247–1743 h), respectively. Party composition was documented across 1210 1‐h time blocks (hereafter referred to as OTBs), comprising 462 OTBs for males and 748 OTBs for females. The mean observation time per OTB was 51.8 min, ranging from 0.2 to 60.0 min.

### 2.3 Definition of Copulation

Copulation refers to a male mounting a female with penile‐vaginal intromission and pelvic thrusting. Because ejaculation is frequently visually imperceptible, these interactions were recorded as copulations regardless of confirmed sperm expulsion (Hashimoto 1997; Ryu et al. 2025). We recorded all observable copulatory interactions between adolescent or adult individuals ad libitum. To avoid inflating copulation rates, we treated successive copulations by the same dyad within a 10‐min window as a single event.

In total, we recorded 268 copulations (19–38 events per male): 67 involving the focal male, 67 involving the focal female, and 134 ad libitum observations involving non‐focal individuals. Visible sperm expulsion from the vagina immediately post‐copulation occurred in 17 of these 268 events (6.3%). Copulations were observed from all adult and adolescent males except for one older male, TW (estimated at 51 years old). Because TW has exhibited no erections and copulatory behavior since at least 2015 (K. Toda, personal observation), his focal data (38.5 h and 42 OTBs) were excluded from the analyses.

### 2.4 Definition of Aggression

Aggression here refers to directed interactions, including contact aggression (e.g., punching, kicking, pushing, tackling, and biting) and threat displays (e.g., chasing, charging, charging while branch‐dragging, and branch‐shaking) (Nishida et al. 1999). Undirected displays lacking a clear target were not classified as aggression. We recorded all observable aggressive interactions among adults and adolescents, noting the target's response—namely, submission (e.g., fleeing, grinning, and screaming) or retaliation. To avoid inflating counts, we treated successive aggressive interactions between the same dyad within 10 min as a single aggression event.

In total, we recorded 297 dyadic aggressive interactions: 124 involving the focal male, 51 involving the focal female, and 122 ad libitum observations involving non‐focal subjects. These comprised 138 male–male (46.5%), 81 male‐to‐female (27.3%), 62 female‐to‐male (20.9%), and 16 female–female (5.4%) interactions. Contact aggression occurred in 95 events (32.0%), initiated by males in 43 (45.2%) and females in 52 (54.7%). Targets exhibited submissive responses in 201 events (67.7%), enabling the determination of winners and losers.

### 2.5 Evaluation of Male Dominance Ranks

To infer the E1 group's dominance hierarchy, we calculated David's scores (David 1987) from an interaction matrix of 201 dyadic aggressive interactions identified with winners and losers. This calculation utilized dyadic dominance indices that incorporate win‐loss proportions and correct for unequal interaction opportunities (de Vries 1998). Although our primary aim was to evaluate male dominance rank, we included both sexes in a single matrix because male bonobos are codominant with females (Furuichi 1997; Surbeck and Hohmann 2013). We computed these scores for 12 males (Table 1) and 20 females (Table 2) using the *DS* function in the *EloRating* package (Neumann and Kulik 2024) in R version 4.4.2 (R Core Team 2024). Using the *steepness* and *h.index* functions within the same package, we verified that both hierarchy steepness (de Vries et al. 2006) and the modified Landau's linearity index (de Vries 1995) were significantly greater than chance based on randomized matrices (one‐tailed *p* = 0.001 and *p* = 0.020, respectively).

### 2.6 Assessment of Sexual Swelling States

We recorded daily variation in the sexual swelling states of female bonobos in the E1 group by systematically scoring the size and firmness of the sexual skin. Following established protocols (Furuichi 1987; Ryu et al. 2015), we assigned each female a score of 1 (no swelling), 2 (intermediate swelling), or 3 (maximal swelling) (Ryu et al. 2025). Score 1 denoted the minimum sexual skin size. Because size alone did not always clearly differentiate scores 2 and 3, firmness and shininess were additionally assessed to distinguish between these categories. We defined detumescence day as the first day the score shifted from 3 to 2, marked by a loss of firmness and shininess.

To minimize observation error and scoring bias, researchers and trained assistants discussed and reached a consensus on each female's swelling score during daily evening meetings. Throughout the study period, the mean number of maximally tumescent females observed per day was 4.5 ± 2.4 (range: 0–10), comprising 3.1 ± 1.8 adults (range: 0–7) and 1.4 ± 1.0 adolescents (range: 0–3).

We defined the maximal swelling phase (MSP) as the interval from the first day a female scored 3 to the last consecutive day she scored 3, immediately preceding detumescence. To address scoring gaps caused by absences or missed observations, we interpolated identical scores across gaps of ≤ 3 days. If a score dropped from 3 to 2 but returned to 3 within 4 days, the intervening score‐2 days were considered as part of a continuous MSP, consistent with previous studies (Douglas et al. 2016; Ryu et al. 2025). In total, we identified 48 MSPs across 13 adult and 3 adolescent females (Table 2).

### 2.7 Estimation of Female Fertile Windows

The association between ovulation timing and the maximal swelling phase (MSP) in female bonobos has been investigated by measuring urinary or fecal ovarian steroid hormones. While some studies indicate that ovulation occasionally occurs outside the MSP, complicating predictions of the fertile window based on MSP onset or end (Reichert et al. 2002; Douglas et al. 2016), others present evidence that ovulation is concentrated toward the end of the MSP, enabling retrospective estimation of the fertile window from the day of detumescence (Heistermann et al. 1996; Ryu et al. 2025). In primate field studies, the fertile phase is typically defined as a 4‐day window encompassing the day of ovulation and the three preceding days (Deschner et al. 2003; Douglas et al. 2016). This operational definition reflects the short post‐ovulatory viability of the ovum (generally < 24 h) and human fertility studies indicating that most conceptions result from sperm released within 3 days prior to ovulation (though conception possibly occurs with sperm older than 3 days) (Wilcox et al. 1995; Dunson et al. 1999). Applying this criterion to bonobos, Ryu et al. (2025) estimated that the probability of sperm‐ovum overlap (i.e., fertility) remains high from Days 7 to 2 before detumescence, peaking 4–5 days prior, and declines to zero by the day preceding detumescence. Ryu et al. (2025) also demonstrated that male bonobos intensively followed females during the late MSP and largely ceased this behavior post‐detumescence.

The spatial dispersion and fluid party composition inherent to fission‐fusion dynamics preclude the frequent, concurrent hormone sampling necessary to pinpoint ovulation in all females within a free‐ranging group. To overcome this limitation, we estimated the likely fertile window (LFW) using visual assessments of sexual swellings. Adopting the framework validated by Ryu et al. (2025), we operationally defined the LFW as the 6‐day period spanning 7–2 days prior to the end of the MSP in ovulatory cycles. Because visual MSPs do not perfectly align with ovulation and frequently occur during anovulatory cycles (Douglas et al. 2016; Ryu et al. 2025), we applied four exclusion criteria to isolate probable ovulatory cycles for LFW estimation:

1. Early adolescence (< 11 years of age): Female bonobos and chimpanzees experience an extended period of subfecundity post‐menarche due to an immature hypothalamic–pituitary‐ovarian axis (Young and Yerkes 1943; Thompson‐Handler 1990; Wallis 1997; Nishida et al. 2003). Captive bonobos reach menarche at approximately 8.1 ± 0.6 years but experience 4.4 ± 2.6 years of adolescent sterility (Thompson‐Handler 1990), making successful reproduction before age 11 appear rare. Therefore, 11 MSPs from 3 adolescent females younger than 11 years old (Tp, Bc, and Ih) were classified as anovulatory.
2. Early lactation (< 24 months postpartum): In the E1 group, the mean interbirth interval was 57.7 ± 9.6 months (Hashimoto et al. 2022), but recent unpublished data from Wamba include intervals of 31–32 months. Accounting for a 7.9‐month gestation (Furuichi et al. 1998), early conception occurs approximately 23–24 months postpartum, aligning with the earliest hormonally identified ovulation at 24 months postpartum (Hashimoto et al. 2022). Based on this threshold, six MSPs from five females with infants younger than 24 months (Yk, Nv, Ot, Jj, and Aq) were classified as anovulatory.
3. Short MSP duration (< 8 days): At Wamba, ovulatory MSPs (mean: 20.7 ± 6.4 days) are often longer than the general cycle average (mean: 14.0 ± 11.2 days) (Ryu 2017). Therefore, five MSPs lasting < 8 days (i.e., the mean minus 2 SD in the ovulatory MSPs) were classified as anovulatory.
4. Short interval to subsequent MSP (< 9 days): At Wamba, luteal phases—marked by elevated progesterone and detumescence—last 10.6 ± 0.6 days (Ryu 2017). Accordingly, two MSPs followed by an interval of < 9 days to the next MSP onset (i.e., the mean minus 2 SD in the luteal phase lengths) were classified as anovulatory.

Consequently, we classified 20 of the 48 MSPs across nine adult females (No, Ki, Hs, Jk, Sl, Fk, Pf, Db, and Mz) as ovulatory cycles (Table 2). For three of the 20 MSPs, sample gaps obscured the exact day of detumescence. In these cases, we assigned an indeterminate LFW status to the final 6 days of the MSP preceding the gap and classified earlier MSP days as non‐LFW. Copulations with adult or adolescent males occurred at least once during 16 of the 17 determined LFWs.

### 2.8 Data Analyses

All statistical analyses were performed in R version 4.4.2 (R Core Team 2024) via RStudio version 12.0.467 (Posit Team 2024). We fitted generalized linear mixed models (GLMMs) with the *glmer* function in the *lme4* package (Bates et al. 2015). Z‐transformation of all continuous predictors (mean = 0, SD = 1) ensured optimal model convergence and interpretability (Schielzeth 2010). The variables included in each model are described below.

To test the first prediction—that mating success differs little across dominance ranks—we examined the effect of male dominance rank on overall copulation rates. This analysis relied on all‐day focal data from 10 males (345.6 h across 41 days), excluding one old male (TW). Because adult male chimpanzees, particularly high‐ranking individuals, exhibit reduced sexual interest in nulliparous compared to parous females (Muller et al. 2006; Reddy et al. 2021), we tallied each focal male's daily copulations with adult and adolescent females separately. To analyze these counts, we fitted two Poisson GLMMs (log link), incorporating an offset for observation time to adjust for varying focal durations (Zuur et al. 2009). Both models evaluated male rank (range: 1–10) as the primary predictor and included the daily number of non‐mother maximally tumescent females (range: 0–6 adults; 0–4 adolescents) as a control variable to capture fluctuations in mating opportunities. A random intercept for focal male identity accounted for repeated measures within each individual (Bolker et al. 2009).

To test the second prediction—that higher‐ranking males copulate more frequently with LFW females than lower‐ranking males—we examined the effect of female LFW status on the probability of copulation with higher‐ versus lower‐ranking males. All‐day focal data on females included 22 days (194.8 h) during which at least one copulation event was observed. After excluding 1 day (9.0 h) during which the focal female's LFW status was indeterminate, we aggregated daily counts of copulations with high‐ranking males (ranks 1–5) and low‐ranking males (ranks 6–10) for the remaining 21 days. These paired counts served as a two‐column response in a binomial GLMM (logit link) (Zuur et al. 2009). This model evaluated female LFW status (LFW: *N* = 4 days; non‐LFW: *N* = 17) as the primary predictor and incorporated a random intercept for focal female identity. To adjust for male availability, the model also included the observed ratio of high‐ to low‐ranking males as a control predictor.

To complement the test of the second prediction, we also examined the effect of male dominance rank on the proportion of copulations with LFW versus non‐LFW females. This supplementary analysis leveraged all copulation events (*N* = 268) recorded through both focal and ad libitum sampling. Excluding four events involving females of indeterminate LFW status, we aggregated the number of copulations with LFW (*N* = 62 events) and non‐LFW females (*N* = 202 events) for each male. A subsequent binomial GLM (logit link) analyzed these two‐column responses (LFW, non‐LFW), with male rank (range: 1–10) as the primary predictor.

To test the third prediction—that subordinate males selectively target females who are less closely guarded by dominant males as mating partners—we assessed the effect of within‐party male dominance rank on the probability of copulation within each one‐hour time block (OTB) during male focal follows. Specifically, we fitted three separate binomial GLMMs (logit link) to examine whether, within the focal party, (i) higher‐ranking males were more likely to copulate, (ii) higher‐ranking males were more likely to copulate with LFW females, and (iii) whether lower‐ranking males were more likely to copulate with non‐LFW females when LFW females were present.

From an initial 420 OTBs, we excluded 41 in which the focal male was out of sight for > 30 min, as one‐hour party size records in Wamba bonobos stabilize after 30 min of observation (Mulavwa et al. 2008). We also excluded 19 OTBs in which the focal male rested for > 95% of the observation time to eliminate periods of near‐total inactivity. The remaining 360 OTBs contained a mean of 4.3 ± 2.4 males (range: 1–9) and 9.0 ± 4.1 females (range: 0–19), including 2.9 ± 1.9 maximally tumescent non‐mother females (range: 0–9) (hereafter, this subset is referred to simply as “maximally tumescent females”). This subset of maximally tumescent females comprised a mean of 2.5 ± 1.7 non‐LFW and 0.4 ± 0.6 LFW females. At least one maximally tumescent non‐LFW female was present in 328 OTBs (91.1%), and at least one LFW female was present in 117 OTBs (32.5%).

We coded copulation occurrence per OTB as a binary response (1: ≥ 1 copulation involving the focal male; 0: no copulation). Copulations spanning consecutive OTBs defaulted to the earlier OTB based on start time. All models incorporated random intercepts for focal male identity and sampling date to account for repeated measures within each individual and day. Model‐specific datasets and predictions were structured as follows:

1. The first model was fitted to 337 OTBs from 40 focal follows, excluding 23 OTBs that lacked maximally tumescent females. Copulations occurred in 58 of these OTBs. The model evaluated the within‐party rank of the focal male (range: 1–8)—corresponding to the number of higher‐ranking males—as the primary predictor, and the numbers of maximally tumescent females (range: 1–9) and males other than the focal male (range: 0–7) as control predictors.
2. The second model was fitted to 127 OTBs from 18 focal follows, excluding OTBs that lacked LFW females (*N* = 243) or contained at least one female of indeterminate LFW status (*N* = 31). Copulations with LFW females occurred in 10 OTBs. Predictors comprised the within‐party rank of the focal male (range: 1–8), LFW females (range: 1–2), maximally tumescent non‐LFW females (range: 0–7), and other males (range: 0–7).
3. The third model was fitted to 307 OTBs from 38 focal follows, excluding OTBs that lacked maximally tumescent non‐LFW females (*N* = 32) or contained any female of indeterminate LFW status (*N* = 31). Copulations with non‐LFW females occurred in 43 OTBs. This model included the main effects of the within‐party rank of the focal male (range: 1–8), the numbers of maximally tumescent non‐LFW females (range: 1–7), LFW females (range: 0–2), and males other than the focal male (range: 0–7), alongside an interaction term between the focal male rank and the number of LFW females.

For model selection, we compared the goodness of fit of each candidate model containing the predictors of interest with that of the corresponding null model, which retained only the control predictors and random‐effects structure (Bolker et al. 2009), following a null‐hypothesis significance testing framework (Nickerson 2000). We performed model comparisons using simulated likelihood ratio tests based on parametric bootstrap (1000 iterations) implemented with the *simulateLRT* function in the *DHARMa* package (Hartig 2024). Fixed‐effects estimates were interpreted only if the candidate model significantly outperformed the null model. Parametric bootstrap inference (2000 iterations) via the *bootMer* function in the *lme4* package yielded confidence intervals for fixed effects.

For each fitted model, residual diagnostics (normality, dispersion, and outliers), evaluated via the *simulateResiduals* function in the *DHARMa* package (Hartig 2024), showed no violations (Figures [Link], [Link]). Similarly, the *check_autocorrelation* and *check_collinearity* functions in the *performance* package (Lüdecke et al. 2021) confirmed residual independence (minimum *p* = 0.150) and low multicollinearity (maximum VIF = 2.64).

## 3 Results

### 3.1 Descriptive Statistics of the Observed Copulation Events

During male focal follows, maximally tumescent females were present on 38 of 41 focal days (92.7%), with at least one female in the LFW present on 17 days (41.5%). Each focal day contained a mean of 4.1 ± 2.4 maximally tumescent females per day (adults: 2.4 ± 1.9; adolescents: 1.6 ± 1.2). Focal males copulated on 33 of the 38 days (86.8%) when receptive females were available. Across the 10 focal males, the mean copulation rate was 0.20 ± 0.08 events per hour (range: 0.06–0.29), comprising 0.12 ± 0.06 events/h with adult females (range: 0–0.21) and 0.07 ± 0.05 events/h with adolescent females (range: 0–0.19). Of the 83 male–male aggressive interactions involving focal subjects, only five events (6.0%) occurred in direct mating contexts (e.g., during solicitation or immediately post‐copulation).

During female focal follows, the mean copulation rates during LFW (*N* = 4 days) and maximally tumescent non‐LFW (*N* = 21 days) were 0.42 and 0.25 events per hour, respectively. Copulations occurred on all 4 days (100%) for LFW females, 15 of 21 days (71.4%) for maximally tumescent non‐LFW females, and only two of 46 days (4.3%) for non‐maximally tumescent non‐LFW females. Of the 15 copulations with LFW females, high‐ranking males (ranks 1–5) accounted for 13 events (86.7%), with the alpha male participating in five (33.3%). Conversely, of the 51 copulations involving non‐LFW females, high‐ranking males accounted for 21 events (41.2%), with the alpha male participating in just two (3.9%).

Across all focal and ad libitum observations, males directed a mean of 19.6% of their copulations toward LFW females (median: 9.0%; range: 0%–54.8%). The alpha male (KT) and his maternal brothers (KY, NB) achieved the highest proportions of these copulations with LFW females (54.8%, 51.4%, and 35.7%, respectively), substantially exceeding the across‐male average. Their resident mother, the alpha female (Ki), supported her sons in conflicts with non‐sibling individuals on four occasions, including one interception of a competitor's copulation attempt. Furthermore, although the older brother (KT) directed 27 aggressive acts against his younger brother (KY)—making them the most frequently aggressive dyad in this group—Ki intervened in six of these encounters to suppress KT's aggression.

Excluding the alpha female's sons, the third‐ranking male (JO) secured a higher proportion of copulations with LFW females (19.4%) than did the four lower‐ranking males with resident mothers (HC, SE, YD, NI; range: 3.7%–10.0%). Notably, JO achieved this relative success despite his mother (Jk) never providing observable agonistic support. Furthermore, the two males without resident mothers (DI, GC) were largely excluded from these mating opportunities, securing only one and zero copulations with LFW females, respectively.

Visible sperm expulsion occurred in 17 of 268 observed copulations (6.3%). After excluding four copulations involving females of indeterminate LFW status (none of which resulted in visible sperm expulsion), the rate of sperm expulsion confirmation was nearly three times as high during copulations with LFW females (12.9%, 8 of 62 events) compared to non‐LFW females (4.5%, 9 of 202). This difference was statistically significant (Fisher's exact test: *p* = 0.033).

### 3.2 Effects of Male Dominance Rank on Overall Copulation Rates

Male dominance rank did not significantly improve model fit over the respective null models for copulation counts with either adult (Poisson GLMM: log‐likelihood ratio = 0.19, *p* = 0.503; see Table S1A) or adolescent females (log‐likelihood ratio = 0.13, *p* = 0.623; see Table S1B). These results indicate that overall copulation rates are independent of male dominance status, supporting our first prediction that mating opportunities are broadly comparable across ranks.

### 3.3 Rank‐Biased Access to Females in the LFW

**FIGURE 1:**
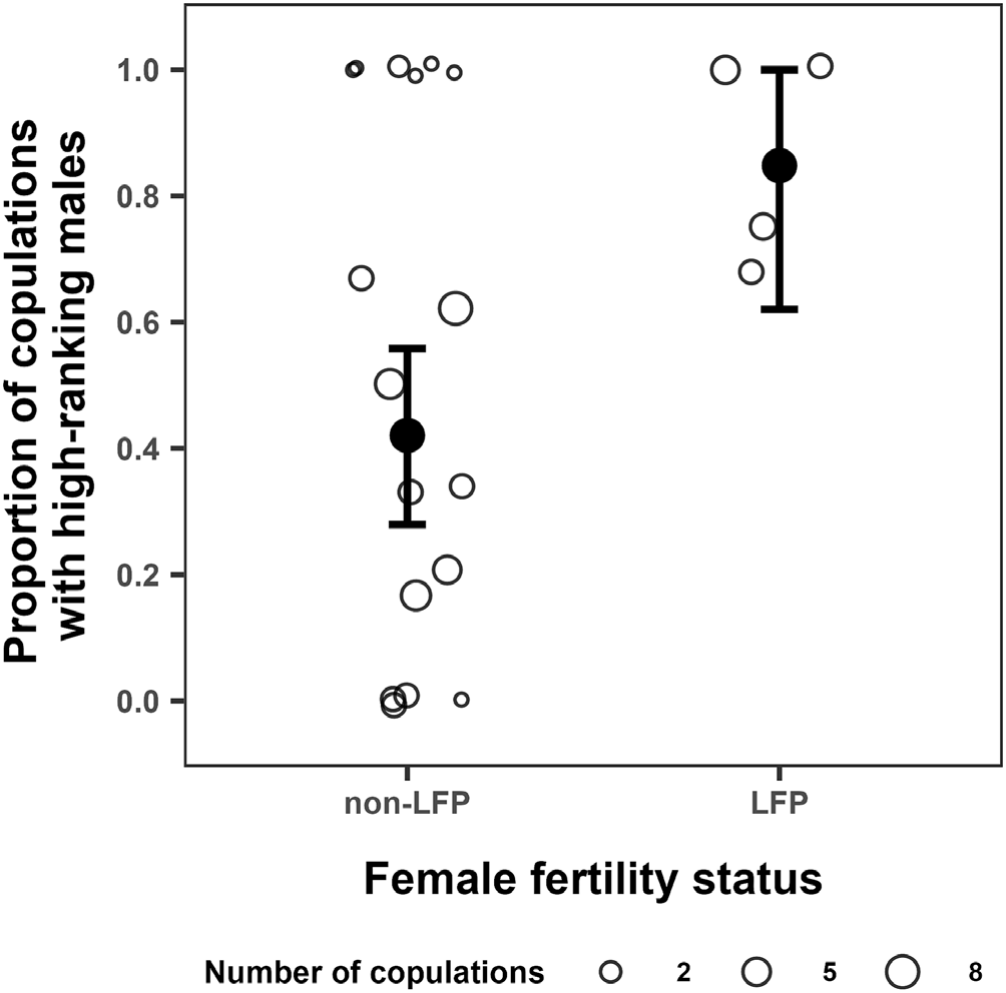
Comparison of the proportion of copulations with high‐ranking males between females in the likely fertile window (LFW) and non‐LFW females. Open circles represent observed proportions for each female focal follow, with circle size indicating the total number of copulations. Filled circles denote model‐estimated means, and vertical error bars indicate confidence intervals.

**FIGURE 2:**
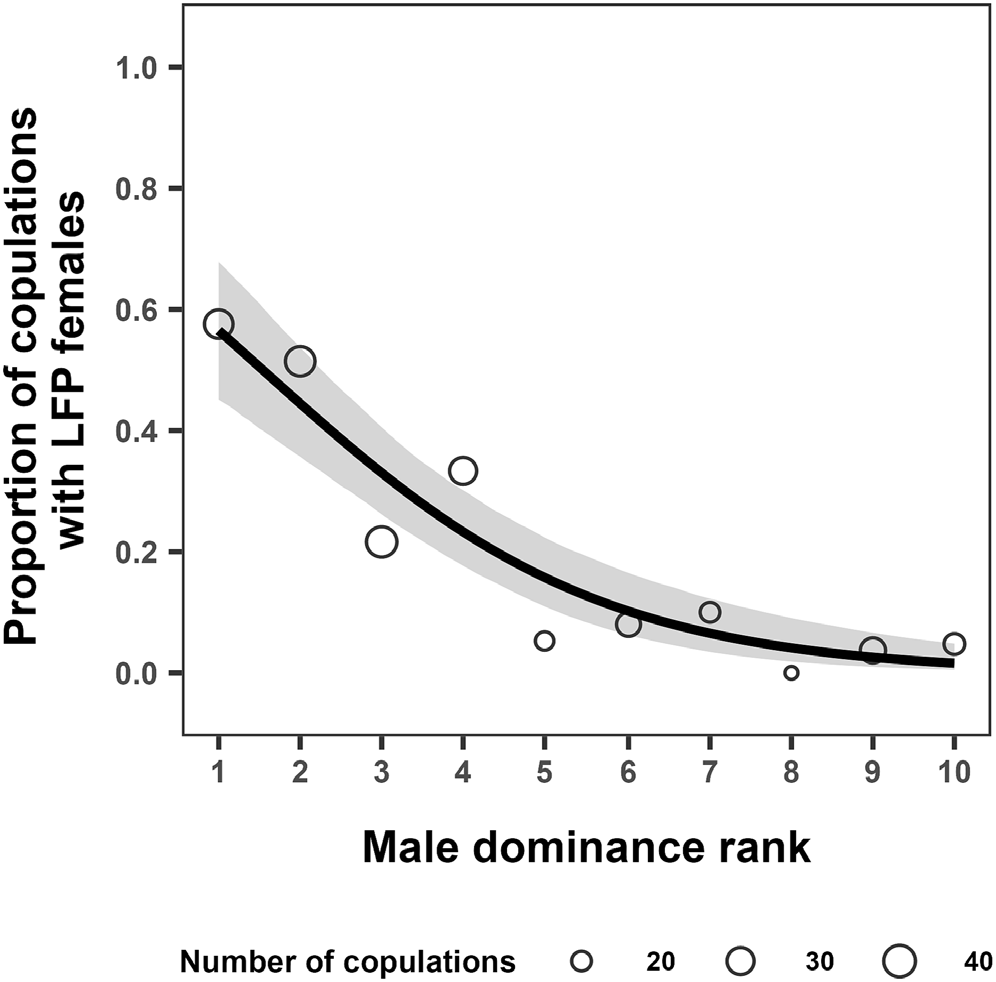
Relationship between male dominance rank and proportion of copulations involving females in the likely fertile window (LFW). Male dominance rank is ordered such that lower values indicate higher‐ranking males and higher values indicate lower‐ranking males. Open circles represent observed proportions for each male, with circle size indicating the total number of copulations. The solid line shows the model‐predicted relationship, and the shaded area represents the 95% confidence interval.

Female LFW status significantly improved model fit over the null model (Binomial GLMM: log‐likelihood ratio = 3.6, *p* = 0.010), revealing that LFW females were more likely to copulate with high‐ranking males than were non‐LFW females (Table 3A, Figure 1). An additional male‐based analysis across all copulation events with estimable female LFW status corroborated this pattern. Male dominance rank significantly improved model fit over the null model (Binomial GLM: log‐likelihood ratio = 28.3, *p* < 0.001), demonstrating that higher‐ranking males directed a greater proportion of their copulations toward LFW females than did lower‐ranking males (Table 3B, Figure 2). Together, these findings support the second prediction that dominant males secure priority access to fertile females.

**TABLE 3:** Parameter estimates for the models that significantly improved fit over their corresponding null models.

Models	Responses	Predictors	Estimate	SE	*Z*	*p*
*A*: Binomial GLMM (Figure 1)	Proportion of copulations with high‐ versus low‐ranking males	(Intercept)	−0.32	0.29	−1.11	0.266
		Female LFW status (yes, no)	2.04	0.83	2.46	0.014*
		Ratio of numbers of high‐ versus low‐ranking males observed	0.27	0.27	1.02	0.307
*B*: Binomial GLM (Figure 2)	Proportion of copulations with LFW versus non‐LFW females	(Intercept)	−1.96	0.25	−7.77	< 0.001
		Male rank (1–10)	−1.47	0.24	−6.02	< 0.001***
*C*: Binomial GLMM (Figure 3)	Probability of copulations with LFW females within OTBs	(Intercept)	−3.37	0.65	−5.21	< 0.001
		Within‐party male rank (1–8)	−1.59	0.68	−2.35	0.019*
		Number of mature males other than the focal male (0–7)	−0.55	0.45	−1.25	0.212
		Number of LFW females (1–2)	0.82	0.40	2.04	0.041*
		Number of non‐LFW females (0–7)	−0.16	0.50	0.32	0.747
*D*: Binomial GLMM (Figure 4)	Probability of copulations with non‐LFW females within OTBs	(Intercept)	−2.12	0.22	−9.51	< 0.001
		Within‐party male rank (1–8)	0.15	0.23	0.62	0.537
		Number of mature males other than the focal male (0–7)	−0.39	0.27	−1.47	0.141
		Number of LFW females (0–2)	−0.66	0.26	−2.55	0.011**
		Number of non‐LFW females (1–7)	0.74	0.24	3.14	0.002***
		Interaction between within‐party male rank and the number of LFW females	0.57	0.23	2.45	0.014**

*Note:* All continuous predictors are *z* transformed (mean = 0 and SD = 1). Asterisks indicate significance levels, *: *p* < 0.05; **: *p* < 0.01; ***: *p* < 0.001.

Abbreviations: GLM, generalized linear model; GLMM, generalized linear mixed model; LFW, likely fertile window; OTB, one‐hour time block.

### 3.4 Effects of Within‐Party Male Dominance Ranks on Mating Partners

Within‐party male dominance rank did not significantly improve model fit over the null model for the overall occurrence of copulations within one‐hour time blocks (OTBs) (Binomial GLMM: log‐likelihood ratio = 0.14, *p* = 0.598; see Table S1C). This independence of copulation probability from local rank contexts aligns with the first prediction.

**FIGURE 3:**
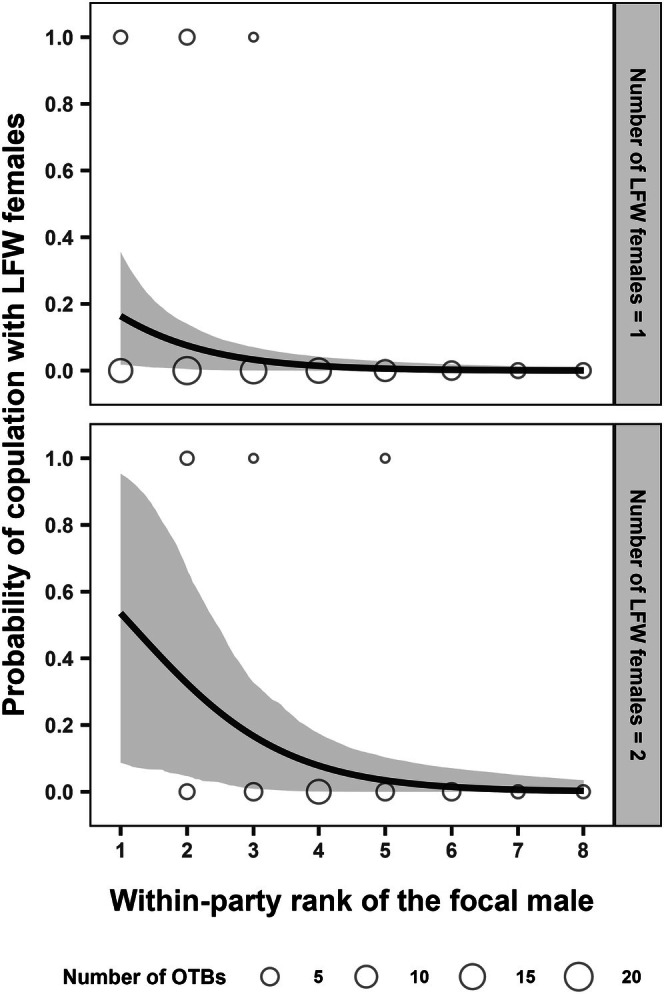
Relationship between within‐party male dominance rank and the probability of copulation with females in the likely fertile window (LFW). Predicted copulation probabilities are shown separately for one‐hour time blocks (OTBs) containing one (top panel) or two (bottom panel) females in the LFW. The size of open circles indicates the number of OTBs. Solid lines show model‐predicted probabilities, and shaded areas represent 95% confidence intervals on the probability scale.

Conversely, within‐party male rank significantly improved model fit for copulations with LFW females (log‐likelihood ratio = 3.76, *p* = 0.007). When LFW females were present, higher‐ranking males were more likely than lower‐ranking males to copulate with them (Table 3C; Figure 3), consistent with the second prediction.

**FIGURE 4:**
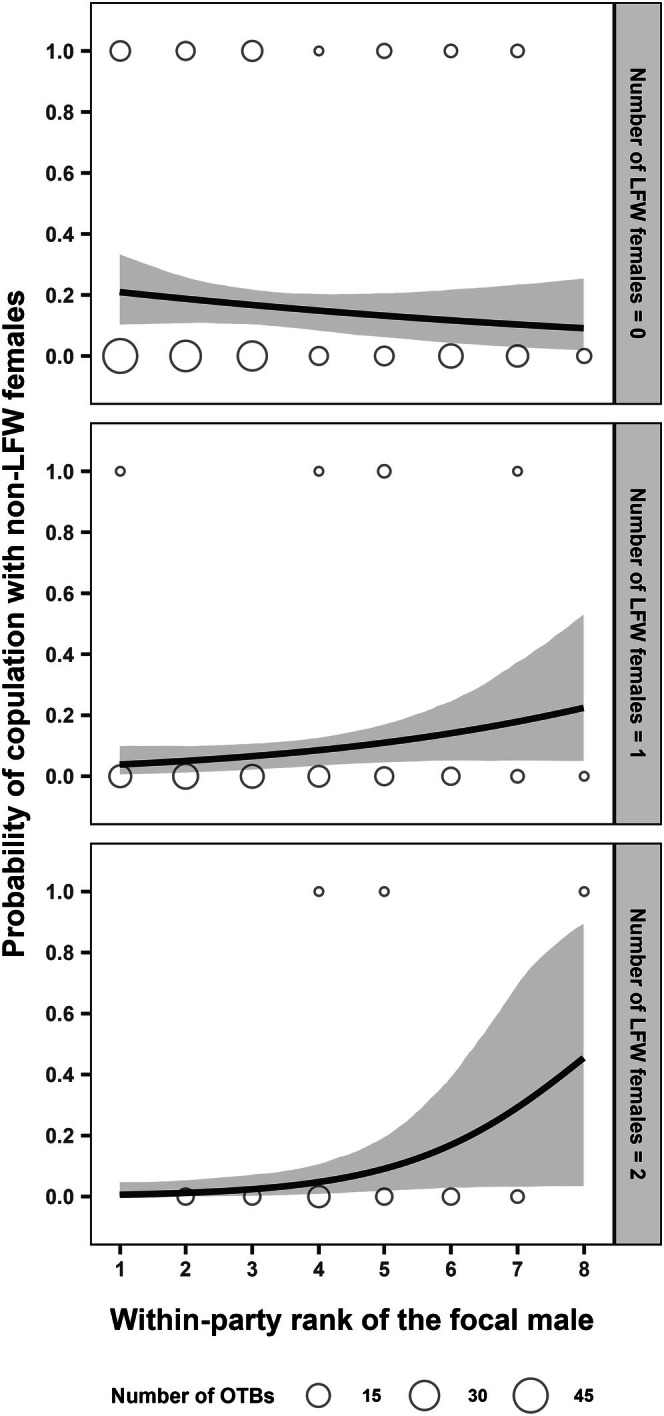
Relationship between within‐party male dominance rank and the probability of copulation with females outside the likely fertile window (LFW), changing with the number of LFW females. Predicted copulation probabilities are shown separately for one‐hour time blocks (OTBs) containing zero (top panel), one (middle panel), or two (bottom panel) females in the LFW. The size of open circles indicates the number of OTBs. Solid lines show model‐predicted probabilities, and shaded areas represent 95% confidence intervals on the probability scale.

Furthermore, the interaction between within‐party male rank and the number of LFW females significantly improved model fit for copulations with non‐LFW females (log‐likelihood ratio = 2.99, *p* = 0.027). This significant interaction indicates a dynamic shift in mating patterns: as the number of LFW females increased from zero to two, the probability of copulating with non‐LFW females shifted disproportionately toward lower‐ranking males (Table 3D; Figure 4). This conditional shift supports the third prediction that subordinate males redirect mating effort toward females with lower conception probabilities under competitive constraints.

## 4 Discussion

### 4.1 Comparable Copulation Rates Across Male Dominance Ranks

All‐day focal follows of male bonobos revealed no detectable relationship between dominance rank and overall copulation rates with either adult or adolescent females. Copulations occurred on 80.5% (33 of 41) of focal days, with males across the entire hierarchy engaging in copulation on more than half of their respective focal days (averaging approximately two events per day). Similarly, within‐party male rank had little effect on copulation probability when female fertility status was not accounted for. The frequent concurrent availability of multiple maximally tumescent females (4.1 ± 2.4 females per day) likely dispersed male mating effort across receptive females and mitigated the monopolization of mating opportunities by dominant males. This interpretation aligns with the priority‐of‐access (PoA) model, which posits that a single male cannot simultaneously control access to multiple females (S. A. Altmann 1962).

Our findings contrast with prior reports of rank‐biased copulation rates favoring dominant males (Kano 1996; Hohmann and Fruth 2003; Surbeck et al. 2011; Yokoyama and Furuichi 2022; Ryu et al. 2025). Methodological differences likely explain this discrepancy. Previous studies relying on provisioning or short‐duration focal follows (10–60 min) often overrepresent centrally positioned individuals and larger parties, thereby missing copulations occurring at the periphery or during small‐party ranging (J. Altmann 1974; Drickamer 1974). Capturing these peripheral copulation events is critical as subordinate males may employ opportunistic tactics to pursue mating opportunities while avoiding interference from dominant males (Furuichi and Ihobe 1994; Kano 1996; this study). Thus, all‐day focal follows provide less biased estimates of rank‐related mating activity. Nevertheless, because our inference is based on 6 months of observations in a single group, assessing the generality of the rank‐independent copulation rates requires multi‐group and long‐term datasets using comparable all‐day protocols.

### 4.2 Rank‐Biased Access to Females in the Likely Fertile Window and Predictability of Ovulation From Graded Signals

Despite the absence of rank effects on overall male copulation rates, high‐ranking males secured a disproportionate share of copulations with females in the likely fertile window (LFW), based on both female‐based focal follows and ad libitum records. This pattern is consistent with genetic evidence for pronounced male reproductive skew in bonobos (Surbeck, Langergraber, et al. 2017; Ishizuka et al. 2018; Mouginot et al. 2023) and with behavioral and hormonal evidence that males can extract usable information about ovulation timing from graded swelling cues and concentrate mating effort in periovulatory periods (Ryu et al. 2025). Although direct disruption of copulations by dominant males was infrequent in our observations, dominants may nonetheless have constrained subordinates' access to LFW females through close following and positional control. Comparable patterns have been widely reported in other non‐human primates in which females remain receptive across extended periods that include infertile days, yet male mating effort is biased toward the periovulatory phase (e.g., chimpanzees: Deschner et al. 2004; mandrills, *Mandrillus sphinx* : Setchell et al. 2005; savannah baboons, *Papio cynocephalus* : Alberts et al. 2006; sifakas, *Propithecus verreauxi* : Mass et al. 2009; rhesus macaques: Dubuc et al. 2012; crested macaques, *Macaca nigra* : Higham et al. 2021). Collectively, these findings underscore that the pronounced reproductive skew in male bonobos is driven primarily by rank‐biased access to fertile females rather than by total mating frequency.

In bonobos, maternal presence and dominance rank significantly enhance sons' reproductive success by conferring competitive advantages through agonistic support and facilitating mating access (Kano 1996; Furuichi 1997; Surbeck et al. 2011; Surbeck et al. 2019; Yokoyama and Furuichi 2022; Shibata and Furuichi 2024). Our observation that all three sons (KT, KY, and NB) of the alpha female (Ki) secured substantially higher proportions of copulations with LFW females accords with the well‐documented pattern in this species. In stark contrast to these strong mother‐son social bonds, males rarely form coalitions with each other (Furuichi and Ihobe 1994; Surbeck and Hohmann 2013; Shibata and Furuichi 2024). In our study, the maternal brothers, especially KT and KY, instead exhibited a consistently hostile relationship. The concession model posits that dominant males permit subordinates to reproduce in exchange for crucial social benefits (e.g., chimpanzees: Duffy et al. 2007; Bray et al. 2016; gelada baboons, *Theropithecus gelada* : Snyder‐Mackler et al. 2012; chacma baboons, *Papio ursinus* : Henzi et al. 2010; red‐fronted lemurs, *Eulemur fulvus* : Port et al. 2010; lions, *Panthera leo* : Packer et al. 1991). This study provides further evidence for the absence of male–male cooperation—even among maternal brothers—suggesting no inherent need for reproductive concessions. Therefore, the pronounced reproductive skew among male bonobos is likely reinforced by both the strong social influence of mothers and a lack of male–male alliances.

Given the energetic costs of ejaculate production (Dewsbury 1982; Thomsen et al. 2006; Parker 2016), males are expected to strategically allocate mating effort by prioritizing periovulatory females and minimizing investment in non‐conceptive copulations (e.g., Aujard et al. 1998; Li et al. 2007; Heistermann et al. 2008; Garcia et al. 2009). In our study, however, even the alpha male engaged in copulations with non‐LFW females during nearly half of his copulation events, with occasional ejaculations confirmed. This supports the premise that male bonobos assess female fertility probabilistically rather than categorically (Ryu et al. 2025). Nevertheless, higher‐ranking males clearly prioritized access to LFW females when they were present within the party. Furthermore, observed ejaculation rates were significantly higher during copulations with LFW females than during those with non‐LFW females. These patterns indicate that males strategically bias not only their behavioral mating effort but also their physiological investment toward females with higher conception probabilities while maintaining residual investment in those with lower conception probabilities.

In this study, we inferred female fertility from daily swelling scores, operationally defining the LFW based on the empirical relationship between detumescence timing and hormone‐estimated ovulation (Ryu et al. 2025). Applying this proxy revealed a rank‐biased mating pattern congruent with established genetic paternity data in bonobos. While this swelling‐based approach provides a valuable alternative when continuous hormonal sampling across all females is infeasible, it must be interpreted as an approximate indicator—rather than a precise measure—due to inherent variability between swelling patterns and the exact timing of ovulation in this species.

### 4.3 Within‐Party Rank Dynamics and Alternative Mating Tactics by Subordinate Males

Party‐level analyses provide additional insight into how male bonobos allocate mating effort on short temporal scales. Although within‐party male dominance rank did not affect the probability of copulation per se, it was strongly associated with the fertility status of mating partners: higher‐ranking males preferentially copulated with LFW females, while lower‐ranking males were more likely to copulate with non‐LFW females as the number of LFW females increased. Our findings suggest that subordinate males secure mating opportunities by targeting females that are not guarded by dominant males. This alternative tactic of shifting mating targets under competitive constraints can marginally increase subordinates' participation in sperm competition. Furthermore, non‐conceptive copulations might represent a strategic prior investment aimed at securing future mating advantages by developing social bonds with females. In several mammalian species, affiliative or cooperative behaviors toward a given female likely facilitate a male's access to her during future periovulatory periods (e.g., chimpanzees: Gomes and Boesch 2009; Langergraber et al. 2013; Reddy et al. 2021; olive baboons, *Papio anubis* : Smuts 1985; Städele et al. 2019; Barbary macaques, *Macaca sylvanus* : Ménard et al. 2001; Assam macaques, *Macaca assamensis* : Kulik et al. 2012; spotted hyenas, *Crocuta crocuta* : East et al. 2003). If socio‐sexual behaviors serve to cultivate intersexual social bonds in bonobos, maintaining frequent copulations even with females in non‐conceptive phases may represent an adaptive behavioral strategy to enhance future reproductive success.

### 4.4 Implications for Potential Benefits From Prolonged Sexual Signaling

This study adds to the evidence that, while female bonobos remain sexually attractive to males for prolonged periods, ovulatory cues are sufficiently informative to bias males' mating effort toward the fertile window (Ryu et al. 2025), thereby allowing dominant males to heavily skew paternity (Mouginot et al. 2024). This combination does not invalidate the paternity confusion hypothesis. Even when paternity is concentrated among dominant males, frequent non‐conceptive copulations with multiple subordinates can still create sufficient uncertainty about paternity to mitigate male incentives for infanticidal aggression (Palombit 2015). It has been demonstrated in some species that a male's tolerance toward an infant is linked to his past mating history with the mother (e.g., chacma baboons: Palombit et al. 1997; Moscovice et al. 2010; Assam macaques: Ostner et al. 2013), but this pattern has not been examined in bonobos. Beyond paternity confusion, another unresolved question is whether females derive additional social benefits from males through non‐conceptive copulations, such as increased tolerance at feeding sites. Although anecdotal, bonobos reportedly exchange sex for food (Kuroda 1984; Van Krunkelsven et al. 1996; but see Yamamoto 2015). Identifying the adaptive benefits females derive from non‐conceptive sexual interactions remains critical for explaining interspecific variation in the duration and dynamics of sexual receptivity among anthropoid primates.

Because exaggerated sexual swellings impose energetic and immunological costs on female primates (Nunn 1999), the early postpartum resumption of maximal swellings exhibited by female bonobos is theoretically expected to confer adaptive benefits that outweigh these physiological burdens. Beyond mediating male–female mating, maximal swellings serve as a powerful socio‐sexual catalyst that attracts other females, thereby enhancing party‐level gregariousness (Surbeck et al. 2021) and eliciting affiliative interactions among females (Ryu et al. 2015; Anzà et al. 2021). Central to this affiliative network is genito‐genital (GG) rubbing, a same‐sex socio‐sexual behavior specific to female bonobos (Kuroda 1980). Females flexibly use GG‐rubbing to mitigate tension in potentially competitive contexts and foster social tolerance across diverse partners (Furuichi 1989; Hohmann and Fruth 2000; Moscovice et al. 2017, 2019). Stable social bonds among unrelated females underpin the coalitionary networks required to maintain female dominance over males—a social structure that effectively deters infanticide and sexual coercion (Furuichi 2023). Accordingly, prolonged sexual signaling may bring adaptive benefits to females through promoting same‐sex affiliation and cooperation.

## Author Contributions

**Kazuya Toda:** funding acquisition, conceptualization, methodology, data curation, formal analysis, validation, investigation, writing – original draft, project administration, visualization. **Furuichi Takeshi:** supervision, writing – review and editing.

## Funding

This work was supported by Japan Society for the Promotion of Science (21K13670) and Canon Foundation in Europe.

## Ethics Statement

Our field studies of free‐ranging bonobos at Wamba in the Luo Scientific Reserve were approved by the Ministry of Scientific Research and the Center for Research on Ecology and Forestry in the Democratic Republic of Congo. This study adhered to the Animal Research Guidelines of The Graduate University for Advanced Studies (SOKENDAI), Japan, revised on March 23, 2021.

## Supporting information


**Table S1:** Parameter estimates for the models that did not significantly improve fit over their corresponding null models.

**Figure S1:** Demographic changes in the E1 group at Wamba between 1976 and 2023. Data on group composition are missing for the period between 1997 and 2003 due to a lack of observations.

**Figure S2:** Diagnostic plots of simulated scaled residuals from the Poisson GLMM testing the effect of male rank on counts of copulations with adult females.

**Figure S3:** Diagnostic plots of simulated scaled residuals from the Poisson GLMM testing the effect of male rank on counts of copulations with adolescent females.

**Figure S4:** Diagnostic plots of simulated scaled residuals from the binomial GLMM testing the effect of female LFW status on the proportion of copulations with high‐ versus low‐ranking males.

**Figure S5:** Diagnostic plots of simulated scaled residuals from the binomial GLM testing the effect of male rank on the proportion of copulations with LFW versus non‐LFW females.

**Figure S6:** Diagnostic plots of simulated scaled residuals from the binomial GLMM testing the effect of within‐party rank of the focal male on the occurrence of copulations within OTBs.

**Figure S7:** Diagnostic plots of simulated scaled residuals from the binomial GLMM testing the effect of within‐party rank of the focal male on the occurrence of copulations with LFW females within OTBs.

**Figure S8:** Diagnostic plots of simulated scaled residuals from the binomial GLMM testing the interaction between within‐party rank of the focal male and the number of LFW females present on the occurrence of copulations with non‐LFW females within OTBs.

## Data Availability

The data that support the findings of this study are openly available in Dryad at https://doi.org/10.5061/dryad.bg79cnps4.
