# Supplementary material for: Comparable Overall Copulation Rates Yet Rank‐Biased Access to Likely Fertile Females in Male Bonobos at Wamba
Source: Am J Biol Anthropol. 2026 Jul 16;190(3):e70318. doi: 10.1002/ajpa.70318 (PMC13375953; doi:10.1002/ajpa.70318)
Supplement: Supplementary file 1 — Table S1: Parameter estimates for the models that did not significantly improve fit over their corresponding null models. [file AJPA-190-e70318-s002.docx]

**Supporting Information**

**FIGURE S1** Demographic changes in the E1 group at Wamba between 1976 and 2023. Data on group composition are missing for the period between 1997 and 2003 due to a lack of observations

**FIGURE S2** Diagnostic plots of simulated scaled residuals from the Poisson GLMM testing the effect of male rank on counts of copulations with adult females

**FIGURE S3** Diagnostic plots of simulated scaled residuals from the Poisson GLMM testing the effect of male rank on counts of copulations with adolescent females

**FIGURE S4** Diagnostic plots of simulated scaled residuals from the binomial GLMM testing the effect of female LFW status on the proportion of copulations with high- versus low-ranking males

**FIGURE S5** Diagnostic plots of simulated scaled residuals from the binomial GLM testing the effect of male rank on the proportion of copulations with LFW versus non-LFW females

**FIGURE S6** Diagnostic plots of simulated scaled residuals from the binomial GLMM testing the effect of within-party rank of the focal male on the occurrence of copulations within OTBs

**FIGURE S7** Diagnostic plots of simulated scaled residuals from the binomial GLMM testing the effect of within-party rank of the focal male on the occurrence of copulations with LFW females within OTBs

**FIGURE S8** Diagnostic plots of simulated scaled residuals from the binomial GLMM testing the interaction between within-party rank of the focal male and the number of LFW females present on the occurrence of copulations with non-LFW females within OTBs

# **TABLE S1.** Parameter estimates for the models that did not significantly improve fit over their corresponding null models

| **Models** | **Responses** | **Predictors** | **Estimate** | **SE** | ***Z*** | ***P*** |
| --- | --- | --- | --- | --- | --- | --- |
| **A**: Poisson GLMM | Counts of copulations with adult females | (Intercept) | –2.40 | 0.20 | –12.11 | < 0.001 |
|  |  | Male rank (1–10) | –0.10 | 0.17 | –0.61 | 0.541 |
|  |  | Number of maximally tumescent adult females | 0.70 | 0.18 | 3.96 | < 0.001*** |
| **B**: Poisson GLMM | Counts of copulations with adolescent females | (Intercept) | –3.07 | 0.45 | –6.88 | < 0.001 |
|  |  | Male rank (1–10) | –1.47 | 0.24 | –6.02 | 0.611 |
|  |  | Number of maximally tumescent adolescent females | 0.233 | 0.18 | 1.30 | 0.193 |
| **C**: Binomial GLMM | Probability of copulations within OTBs | (Intercept) | –1.65 | 0.15 | –10.71 | < 0.001 |
|  |  | Within-party male rank (1–8) | –0.11 | 0.20 | –0.55 | 0.584 |
|  |  | Number of mature males (0–7) | –0.47 | 0.23 | –2.03 | 0.042* |
|  |  | Number of maximally tumescent females (1–9) | 0.56 | 0.19 | 2.95 | 0.003** |

All continuous predictors are z-transformed (mean = 0 and SD = 1).

Asterisks indicate significance levels, *: *P* < 0.05, **: *P* < 0.01, ***: *P* < 0.001.

GLM = generalized linear model; GLMM = generalized linear mixed model; LFW = likely fertile window; OTB = one-hour time block.
