# Supplementary figures and images for: Comparable Overall Copulation Rates Yet Rank‐Biased Access to Likely Fertile Females in Male Bonobos at Wamba
Source: Am J Biol Anthropol. 2026 Jul 16;190(3):e70318. doi: 10.1002/ajpa.70318 (PMC13375953; doi:10.1002/ajpa.70318)

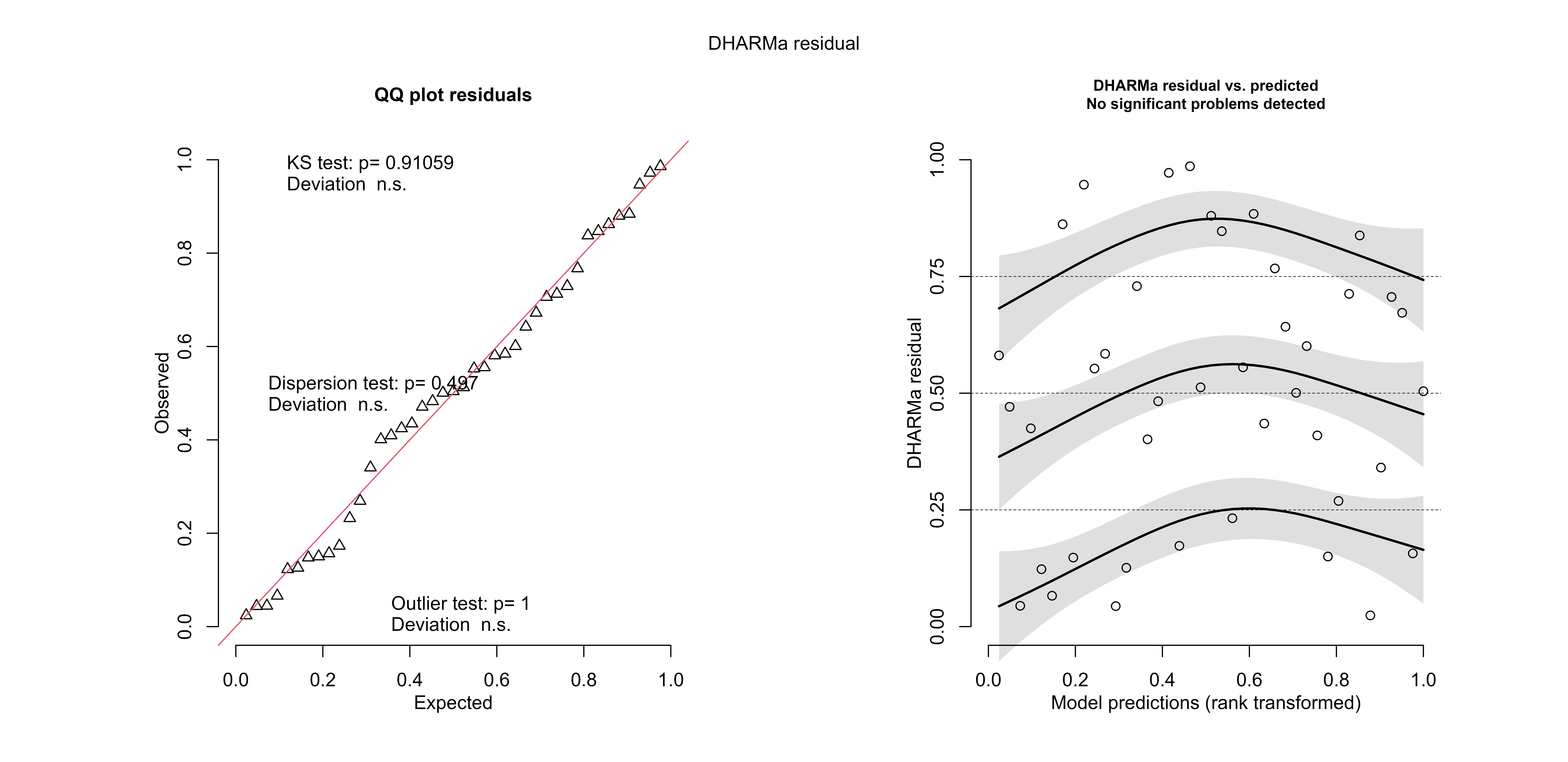

Supplement: Supplementary file 3 — Figure S2: Diagnostic plots of simulated scaled residuals from the Poisson GLMM testing the effect of male rank on counts of copulations with adult females. [file AJPA-190-e70318-s009.png]

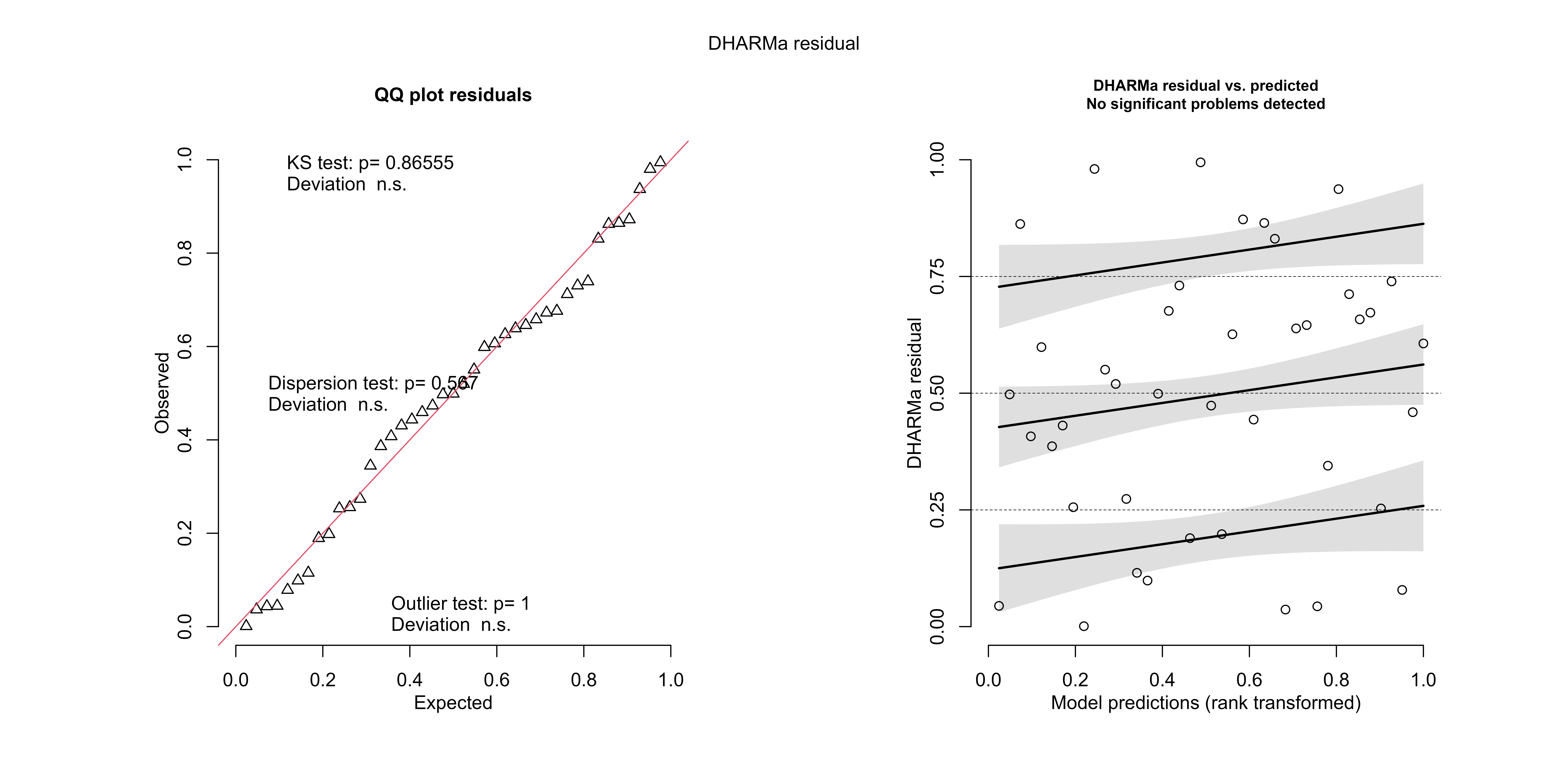

Supplement: Supplementary file 4 — Figure S3: Diagnostic plots of simulated scaled residuals from the Poisson GLMM testing the effect of male rank on counts of copulations with adolescent females. [file AJPA-190-e70318-s007.png]

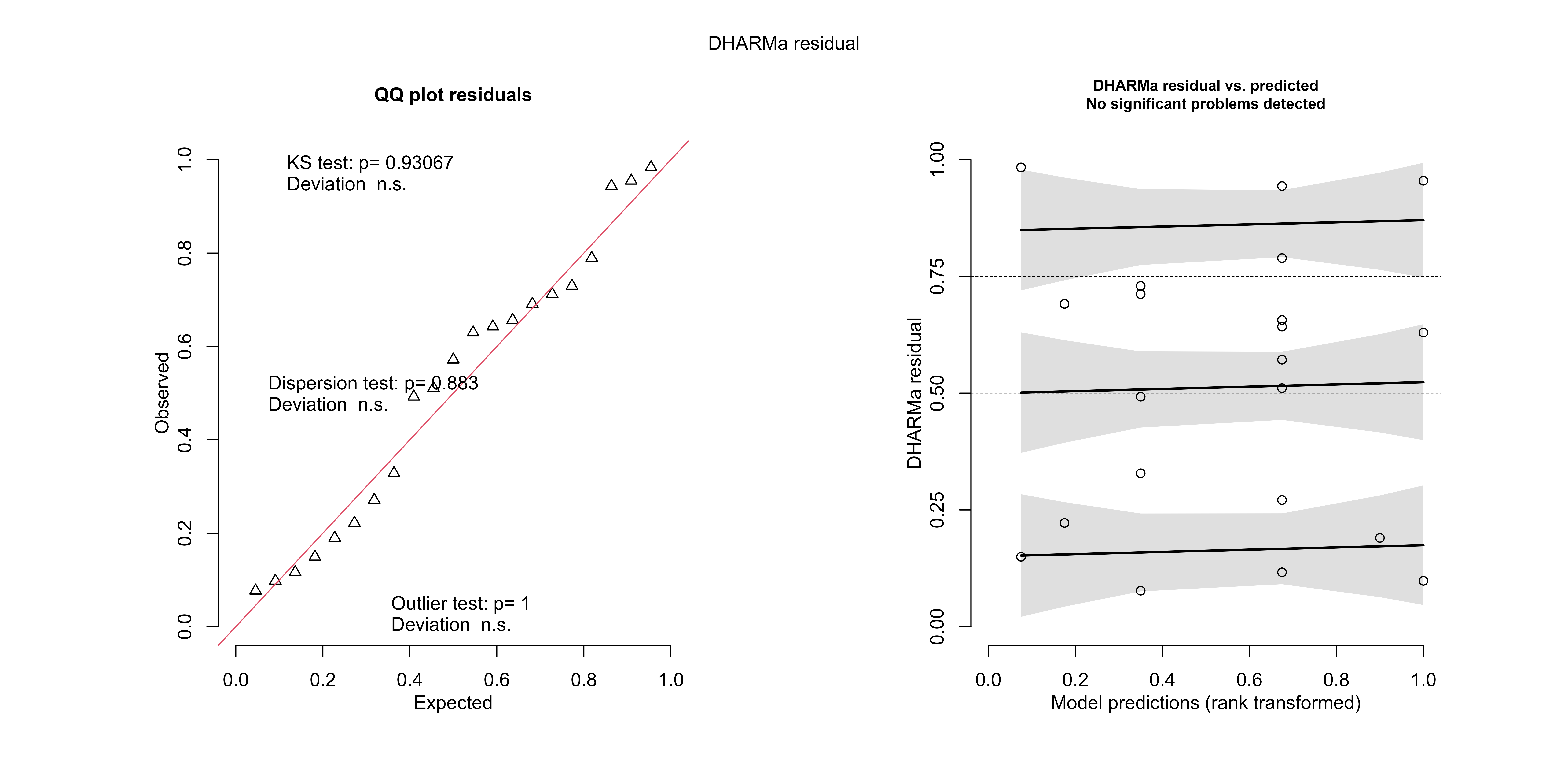

Supplement: Supplementary file 5 — Figure S4: Diagnostic plots of simulated scaled residuals from the binomial GLMM testing the effect of female LFW status on the proportion of copulations with high‐ versus low‐ranking males. [file AJPA-190-e70318-s008.png]

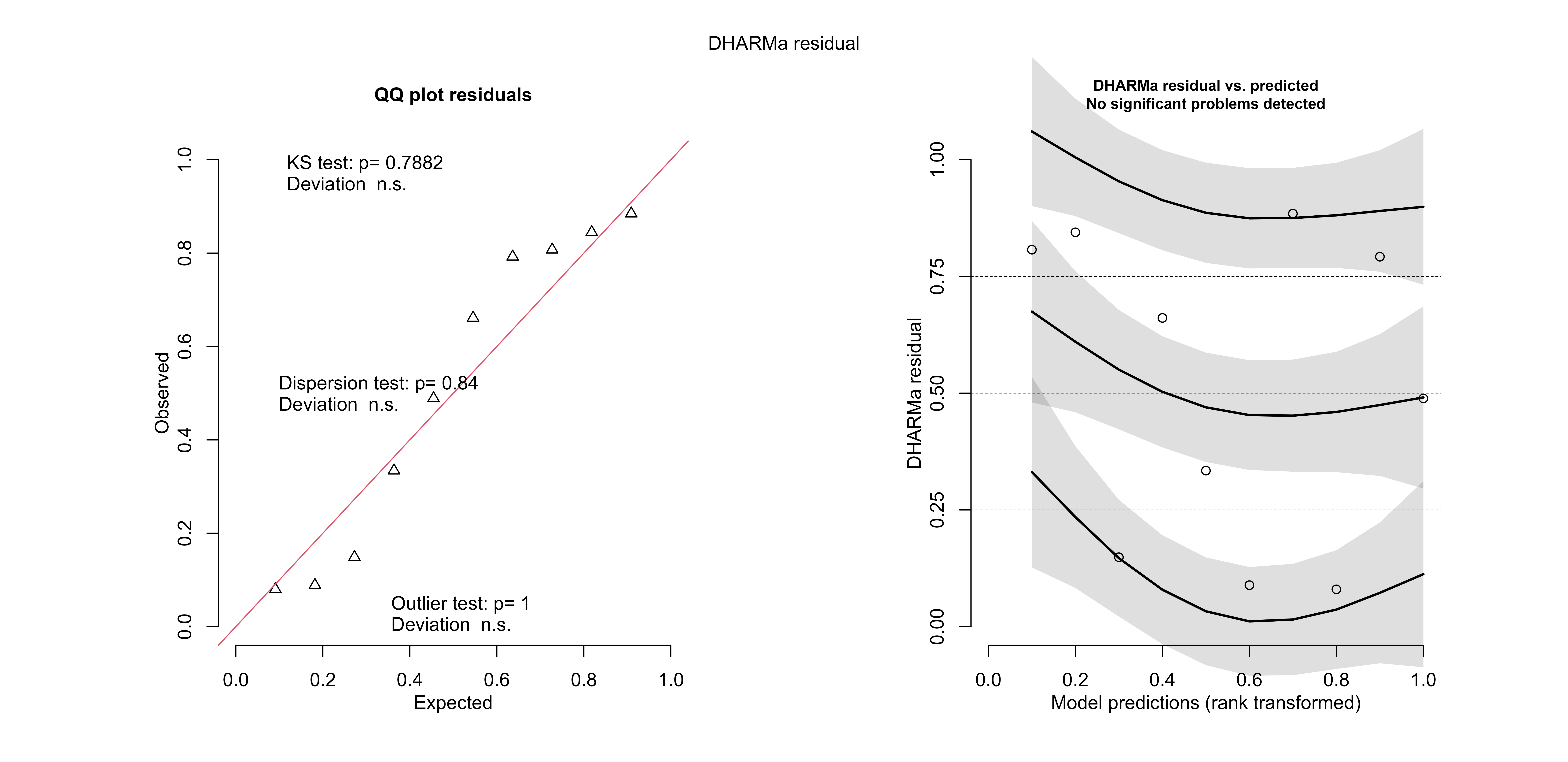

Supplement: Supplementary file 6 — Figure S5: Diagnostic plots of simulated scaled residuals from the binomial GLM testing the effect of male rank on the proportion of copulations with LFW versus non‐LFW females. [file AJPA-190-e70318-s006.png]

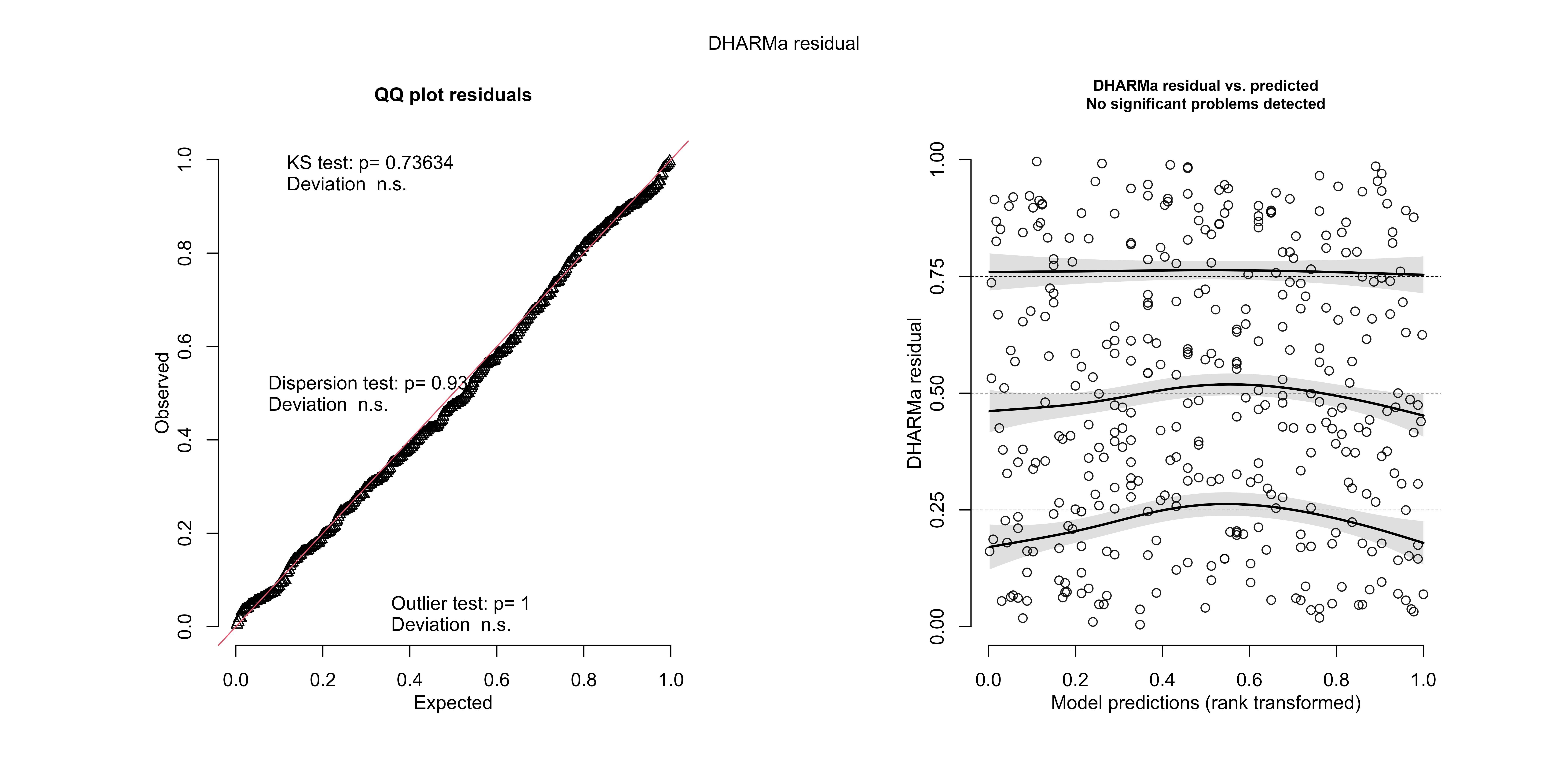

Supplement: Supplementary file 7 — Figure S6: Diagnostic plots of simulated scaled residuals from the binomial GLMM testing the effect of within‐party rank of the focal male on the occurrence of copulations within OTBs. [file AJPA-190-e70318-s004.png]

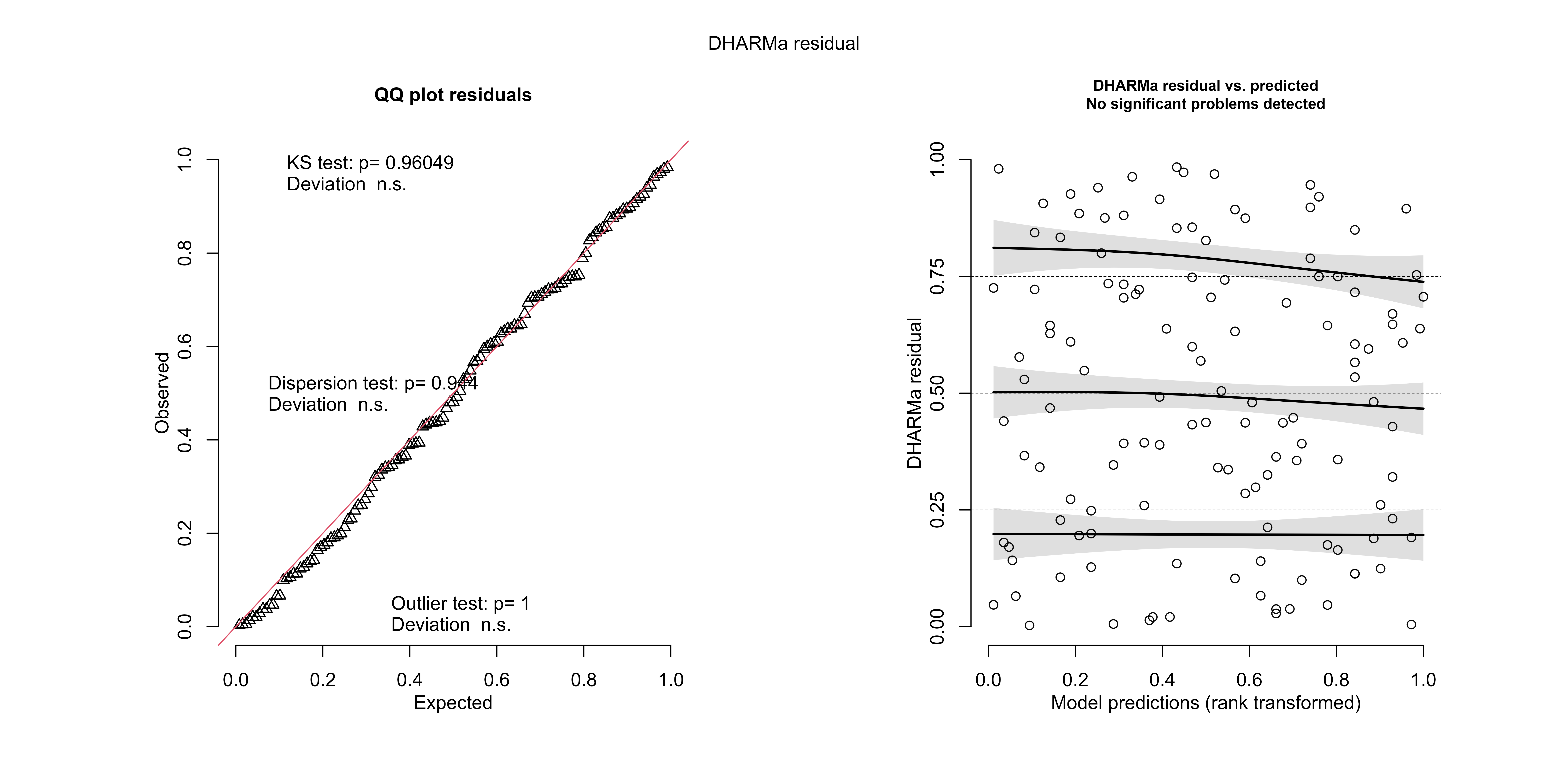

Supplement: Supplementary file 8 — Figure S7: Diagnostic plots of simulated scaled residuals from the binomial GLMM testing the effect of within‐party rank of the focal male on the occurrence of copulations with LFW females within OTBs. [file AJPA-190-e70318-s005.png]

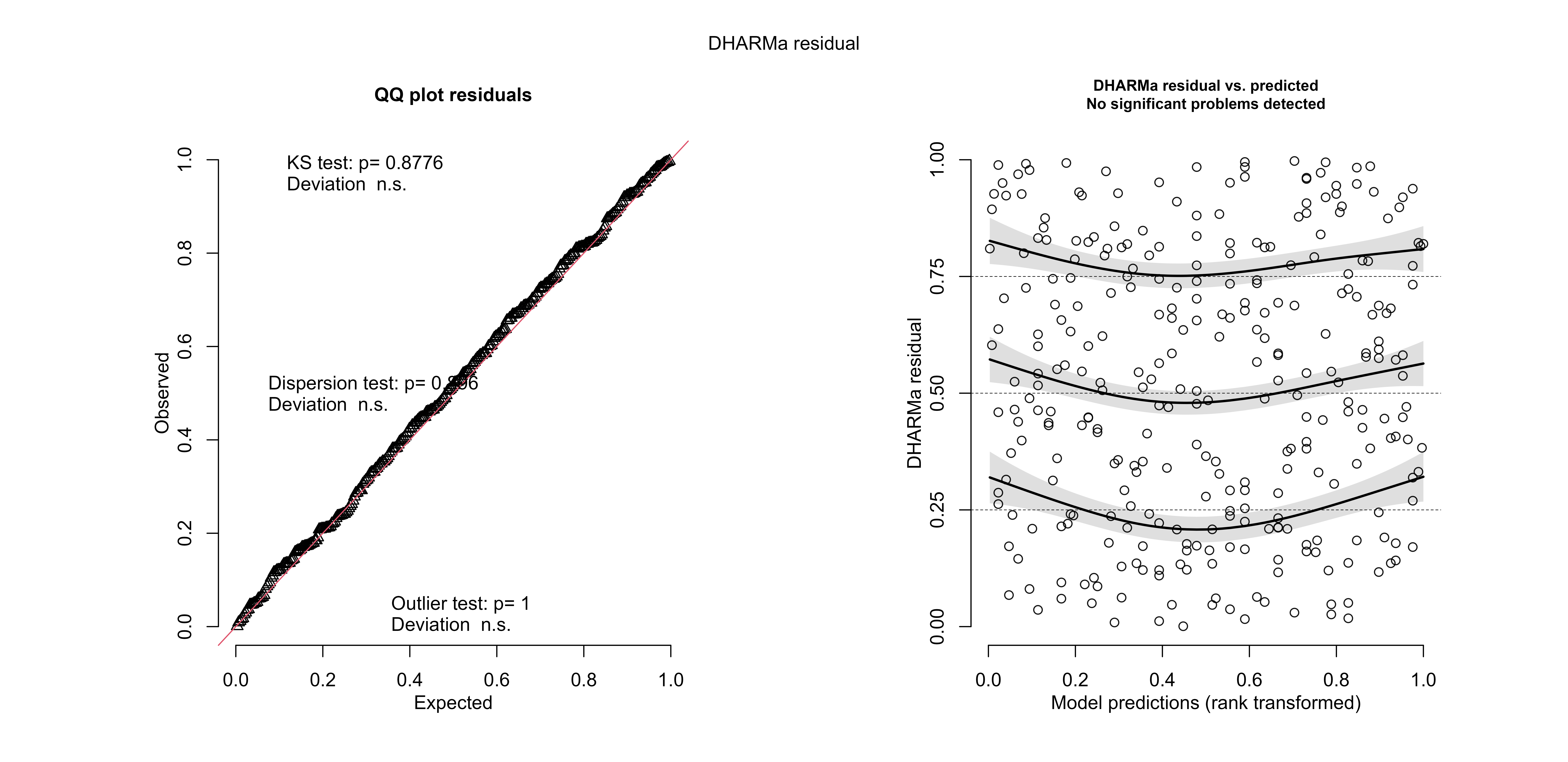

Supplement: Supplementary file 9 — Figure S8: Diagnostic plots of simulated scaled residuals from the binomial GLMM testing the interaction between within‐party rank of the focal male and the number of LFW females present on the occurrence of copulations with non‐LFW females within OTBs. [file AJPA-190-e70318-s001.png]
